# The complete mitochondrial genome sequence and phylogenetic position of *Sinocyclocheilus xiaotunensis* (Cypriniformes: Cyprinidae)

**DOI:** 10.1080/23802359.2021.1914230

**Published:** 2021-05-07

**Authors:** Ke Yu, Yongcai Wang, Miao An, Zhifang Ma, Jian Shao

**Affiliations:** a School of Animal Science, Guizhou University, Guiyang, China; b Agriculture and Rural Affairs Bureau, Zhenfeng County, Guizhou, China

**Keywords:** *Sinocyclocheilus xiaotunensis*, Mitochondrial genome, phylogeny

## Abstract

The complete mitochondrial DNA genome of *Sinocyclocheilus xiaotunensis* was first reported by next-generation sequencing method. The entire length of mitochondrial genome is 16,588 bp and the nucleotide composition was made up of 32.3% A, 25.0% T, 27.2% C, and 15.5% G, indicating an A + T(57.3%) content is greater than C + G(42.7%). The mitogenome is a circular DNA molecule with a D-loop region and contains 22 transfer RNA (tRNA) genes, two ribosomal RNA(rRNA) genes and 13 protein-coding genes.To provide further info on the conserved sequence block observed in the control region of the mitochondrial genome. This info is critical for future application and determination of taxonomic status of this species.

Sinocyclocheilus (Cypriniformes: Cyprinidae) endemic to China and has been listed as endangered species in the IUCN Red List of Threatened Species. However, The fish was collected from Xiaotun Village, Zhenfeng County, Guizhou Province, china (25°50′42″N; 105°60′38″E)、Sancha River, a tributary of Beipanjiang River System and 1165 M above sea level. Through morphological analysis, it is different from *Sinocyclocheilus zhenfengensis* (Liu et al. 2018); through complete mitochondrial genome analysis, it is different from other species, So we named it *Sinocyclocheilus xiaotunensis*. In this study, we first reported the complete mitochondrial genome (mitogenome) of *S.xiaotunensis*, which could provide useful first-hand data for molecular phylogenetics and population genetics studies on this species and its closely related Sinocyclocheilus species. Voucher specimens (gzu20201204) were preserved in 100% ethanol and deposited at zoological museum of the School of Animal Science, Guizhou University.

In terms of morphology, we measured two categories: quantifiable traits and countable traits. In the statistics of the above-mentioned measured growth traits, in order to eliminate the influence on the reliability of the data caused by the size of the fish, we used the head shape measurement value divided by the head length, and the trunk shape measurement value divided by the body Length, the measured value of the tail shape divided by the length of the tail shank.Refer to Table 1 for detailed morphological data. Compared with Liu (2018) (*Sinocyclocheilus zhenfengensis*) collected in the Shuangrufeng Scenic Spot in Zhexiang Town, Zhenfeng County, among the measurable traits, eye diameter/head length, dorsal fin length/body length, pectoral fin length/body length, and pectoral fin length/body length all showed significant differences (*p* < 0.01), and the average values ​​of *S.xiaotunensis* were 0.133, 0.184, 0.207 and 0.301, The average values ​of *S.zhenfengensis* were 0.291, 0.227, 0.247 and 0.337, respectively. In terms of countable traits, the number of dorsal fin spines and gill rakers of *S.xiaotunensis* was more than *S.zhenfengensis*. With the deepening of cave life, the eye diameter of the *S.xiaotunensis* is smaller than the *S.zhenfengensis*, which is related to the light intensity of the living environment(Langecker et al. 1995; Zhou et al. 2009). Not only that the dorsal and pectoral fins of *S.xiaotunensis* became shorter and the number of fin spines increased. This is an adaptive change to the living environment, and its balance function has been further adapted to evolve and it showed obvious difference from *S.zhenfengensis* in morphology.

**Table 1.** Morphological proportion characters of *S. xiaotunensis*.

Character	Holotype	Range (mm)	Character	Holotype	Range (mm)
Number of samples	10				
Body length	85.02	83.87–86.41	Pectoral fin length	17.67	16.79–18.34
Body depth	24.33	22.45–26.4	Anal fin base length	7.65	6.98–8.2
Body width	14.03	12.94–15.03	Dorsal fin base length	11.68	11.02–12.04
Head length	25.31	23.89–26.68	Caudal peduncle depth	9.69	8.33–10.48
Snout length	9.54	8.4–10.14	Caudal peduncle length	15.33	14.36–17.09
Percentage of standard length					
Head height / Head length		0.639	Caudal fin rays		19
Head width / Head length		0.493	Gill rakers		10–11
Interorbital width / Head length		0.292	Lateral line scales		40–42
Eye diameter / Head length		0.133	Scale rows above lateral		11–12
Maxillary barbel length / Head length		0.426	Scale rows below lateral		8–10
Rictal barbel length / Head length		0.461	Pharyngeal teeth		2,3,4–4,3,2
Snout length / Head length		0.376	Dorsal fin rays		III,7–8
Body depth / Body length		0.286	Anal fin rays		III,5–6
Body width / Body length		0.165	Pectoral fin rays		I,14–15
Caudal peduncle depth / Caudal peduncle length		0.632	Pelvic fin rays		I,7–8

Note: Roman symbol(I, III) indicates the number of fin spines.

The complete mitochondrial genome length of *S.xiaotunensis* was 16,588 bp (GenBank accession number MW574480). It consisted of 13 protein-coding genes, two rRNA genes, 22 tRNA genes and one D-loop region (Table 2; Figure 1). The overall base composition of the mitogenome is 32.3% for A, 27.2% for C, 15.5% for G and 25.0% for T. The percentage of G + C content is 42.7%.The gene arrangement and nucleotide composition of the mitogenome of *S.xiaotunensis* were similar to those of other Sinocyclocheilus species (Wu et al. 2010; Chen et al. 2017; Li et al. 2017; Xu et al. 2019). Most mitochondrial genes were encoded on the heavy strand(H-strand), except that the eight tRNA gene and ND6 genes were encoded on the light strand (L-strand). All 13 PCGs except for COI (with a GTG start codon) started with an ATG codon. Six PCGs ended with two types of complete stop codons, TAA(ND1, COI, ND4L, ND5 and ND6)and TAG(ATP8). The remaining PCGs ended with incomplete stop codon, including stop codon T–(ND2, COII, ND3, ND4 and Cytb) and TA-(ATP6 and COIII). The 22 tRNA genes have lengths ranging from 69 to 78 bp. The lengths of 12S and 16S rRNA genes were 955 bp and 1677 bp.The D-loop or control region was located between tRNA-Pro and tRNA-Phe genes with a length of 934 bp. The lengths of COI and Cytb genes were 1551 and 1441 bp, respectively.

**Figure 1.**
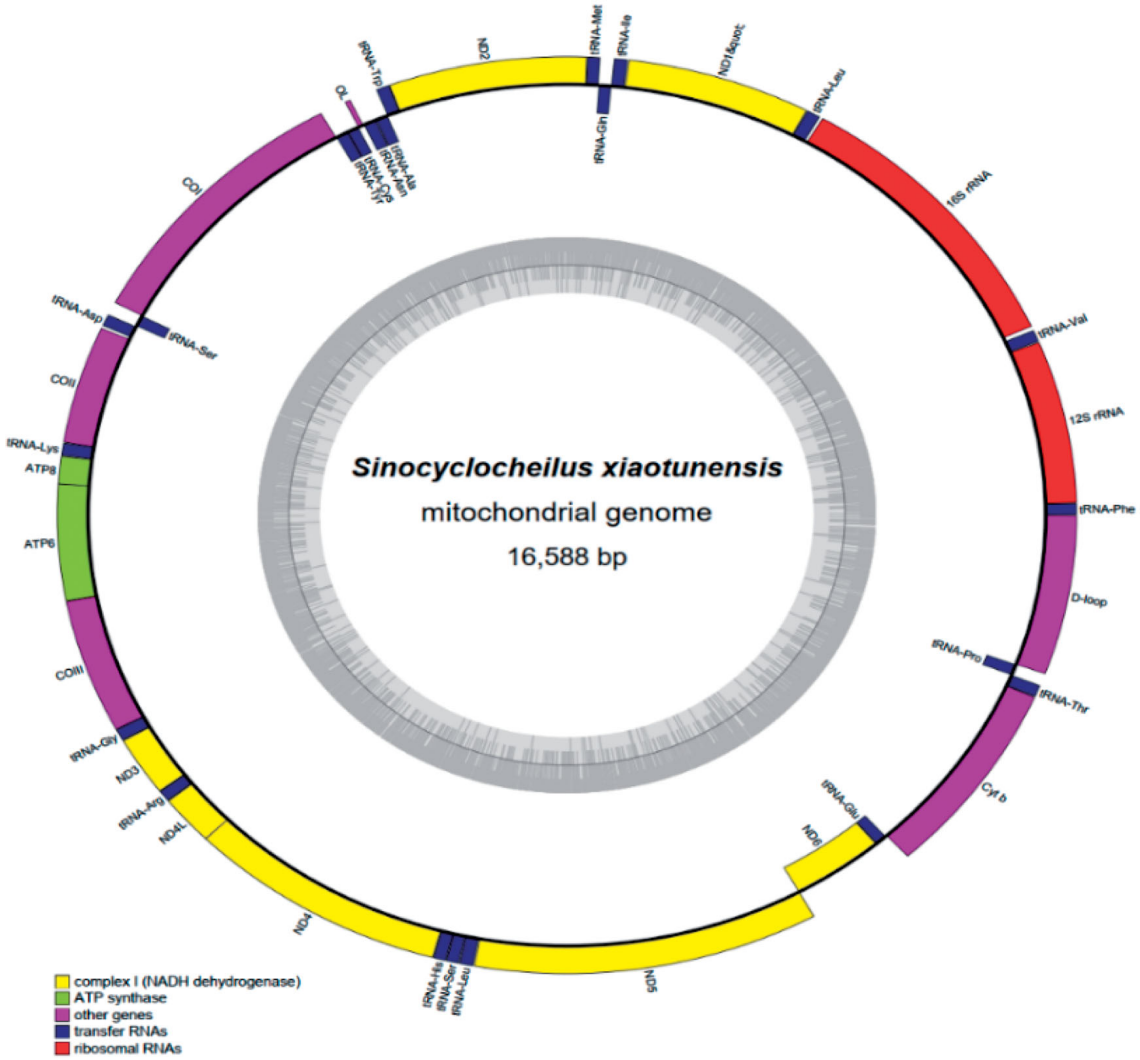
Gene map of *Sinocyclocheilus xiaotunensis* mitogenome. All genes and control region are annotated.

**Figure 2.**
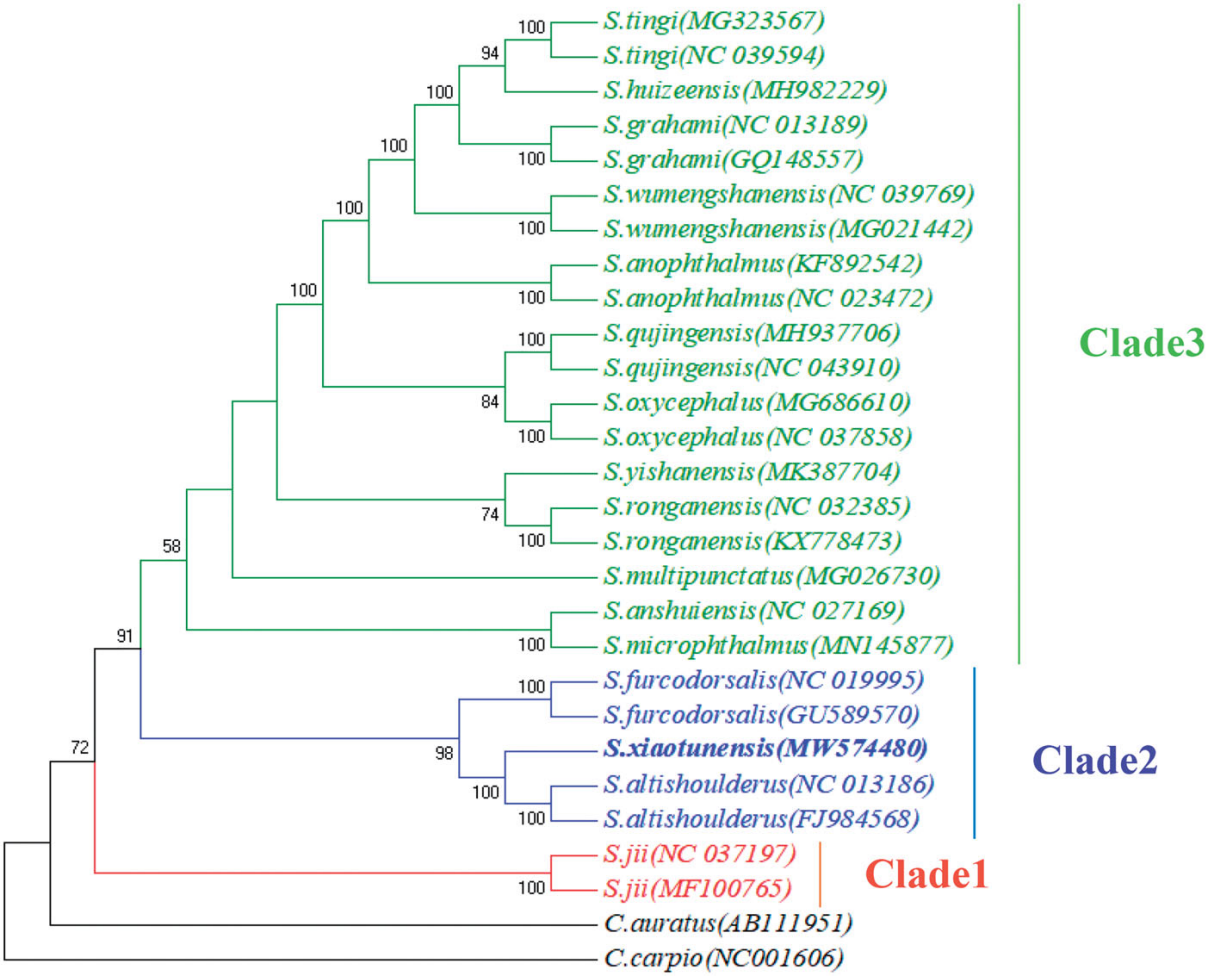
Neighbor-joining phylogenetic tree of the *S. xiaotunensis* and other species based on the complete mitochondrial genome. Numbers on nodes indicate bootstrap support value, based on 1000 replicates.

**Table 2.** Characterization and annotation of *S. xiaotunensis* mitogenome.

Gene	Strand	Position		Length(bp)	Intergrnic nucleotide	Anticodon	Codon	
		From	To				Start	Stop
D-Loop	H	1	934	934	0			
tRNA-phe	H	935	1003	69	0	GAA		
12S rRNA	H	1004	1958	955	0			
tRNA-Val	H	1959	2030	72	0	TAC		
16S rRNA	H	2031	3707	1677	0			
tRNA-Leu(UUR)	H	3708	3785	78	0	TAA		
ND1	H	3786	4760	975	3		ATG	TAA
tRNA-Ile	H	4764	4837	74	−3	GAT		
tRNA-Gln	L	4835	4906	72	0	TTG		
tRNA-Met	H	4907	4975	69	0	CAT		
ND2	H	4976	6022	1047	−2		ATG	T—
tRNA-Trp	H	6021	6093	73	1	TCA		
tRNA-Ala	L	6095	6163	69	1	TGC		
tRNA-Asn	L	6165	6237	73	31	GTT		
tRNA-Cys	L	6269	6337	69	−2	GCA		
tRNA-Tyr	L	6336	6406	71	1	GTA		
COI	H	6408	7958	1551	0		GTG	TAA
tRNA-Ser(UCN)	L	7959	8029	71	4	TGA		
tRNA-Asp	H	8034	8105	72	12	GTC		
COII	H	8118	8808	691	1		ATG	T—
tRNA-Lys	H	8810	8884	75	1	TTT		
ATP8	H	8886	9053	168	−10		ATG	TAG
ATP6	H	9044	9727	684	−1		ATG	TA-
COIII	H	9727	10512	786	−1		ATG	TA-
tRNA-Gly	H	10512	10583	72	0	TCC		
ND3	H	10584	10934	351	−2		ATG	T—
tRNA-Arg	H	10933	11002	70	0	TCG		
ND4L	H	11003	11299	297	−7		ATG	TAA
ND4	H	11293	12673	1381	0		ATG	T—
tRNA-His	H	12674	12743	70	0	GTG		
tRNA-Ser(AGY)	H	12744	12812	69	1	GCT		
tRNA-Leu(CUN)	H	12814	12886	73	3	TAG		
ND5	H	12890	14712	1823	−9		ATG	TAA
ND6	L	14704	15231	528	0		ATG	TAA
tRNA-Glu	L	15232	15300	69	5	TTC		
Cytb	H	15306	16446	1141	0		ATG	T—
tRNA-Thr	H	16447	16518	72	−2	TGT		
tRNA-Pro	L	16517	16588	72	0	TGG		

To determine the phylogenetic position of *S.xiaotunensis*, phylogenetic analyses were conducted based on mitogenome sequences of 25 Sinocyclocheilus species and two outgroup species from GenBank by Neighbor-joining tree (NJ) methods (Tamura et al.^2013^) (Figure 2). The phylogenetic results showed that three clade were observed and S. jii was the most basal species among the Sinocyclocheilus species. Not only that *S.xiaotunensis* was independent with the bootstrap values 100% and had a close relationship with *S.altishoulderus* and *S.furcodorsalis.* In summary, the newly obtained *S.xiaotunensis* extranuclear genomic resource would provide valuable molecular information fundamental to conservation and resource restoration studies on this cyprinid species.

## Data Availability

The genome sequence data that support the findings of this study are openly available in GenBank of NCBI at (https://www.ncbi.nlm.nih.gov/) under the accession no MW574480.

## Museum collector

Miao An, E-mail: gzuam@163.com
